# Gradient Magnetic Field Accelerates Division of *E. coli* Nissle 1917

**DOI:** 10.3390/cells12020315

**Published:** 2023-01-14

**Authors:** Svitlana Gorobets, Oksana Gorobets, Iryna Sharai, Tatyana Polyakova, Vitalii Zablotskii

**Affiliations:** 1 Faculty of Biotechnology and Biotechnics, National Technical University of Ukraine “Igor Sikorsky Kyiv Polytechnic Institute”, 03056 Kyiv, Ukraine; 2 Faculty of Physics and Mathematics, National Technical University of Ukraine “Igor Sikorsky Kyiv Polytechnic Institute”, 03056 Kyiv, Ukraine; 3 Institute of Magnetism of the National Academy of Sciences of Ukraine and Ministry of Education and Science of Ukraine, 03142 Kyiv, Ukraine; 4 Institute of Physics of the Czech Academy of Sciences, Na Slovance 1999/2, 182 00 Prague, Czech Republic; 5 International Magnetobiology Frontier Research Center (iMFRC), Science Island, Hefei 230000, China

**Keywords:** bacterial division, magnetic field, biomagnetic effects, mitosis, intracellular forces

## Abstract

Cell-cycle progression is regulated by numerous intricate endogenous mechanisms, among which intracellular forces and protein motors are central players. Although it seems unlikely that it is possible to speed up this molecular machinery by applying tiny external forces to the cell, we show that magnetic forcing of magnetosensitive bacteria reduces the duration of the mitotic phase. In such bacteria, the coupling of the cell cycle to the splitting of chains of biogenic magnetic nanoparticles (BMNs) provides a biological realization of such forcing. Using a static gradient magnetic field of a special spatial configuration, in probiotic bacteria *E. coli* Nissle 1917, we shortened the duration of the mitotic phase and thereby accelerated cell division. Thus, focused magnetic gradient forces exerted on the BMN chains allowed us to intervene in the processes of division and growth of bacteria. The proposed magnetic-based cell division regulation strategy can improve the efficiency of microbial cell factories and medical applications of magnetosensitive bacteria.

## 1. Introduction

One of the most important and well-known facts of life is that living cells divide. Cell division is a genetically programmed process that controls the whole cell cycle and determines the cell growth rate. The bacterial cell cycle is divided into three stages: (i) the period between cell division (cell birth) and the initiation of chromosome replication (B period); (ii) the period required for chromosome replication (C period); (iii) the time between the end of chromosome replication and completion of cell division (D period) [1]. However, what attributes trigger cell division and determine the duration of each period? Several main factors and their combinations influence the cell cycle: nutrient composition, elapsed time, cell size growth, accumulation of divisome proteins to the threshold, production of ribosomes, DNA and RNA contents, and intracellular mechanical forces. Culture medium composition determines the growth rate of bacteria, and it ultimately dictates the chemical composition of a cell [2]. Additionally, mechanical forces play a critical role in many cellular processes, including cell division, contractility, differentiation, and motility. Mechanical forces drive the segregation of chromosomes into daughter cells, which is one of the most puzzling events during the cell cycle. We assume that it would be possible to accelerate or suspend cell division during mitosis by applying magnetic gradient forces that assist or oppose mechanical intracellular forces during the daughter cells production. Magnetic gradient forces that arise in spatially nonuniform magnetic fields (MFs) can elicit numerous intriguing biological effects in cells such as (i) cytosolic Ca^2+^ level enhancement and induction of actin polymerization [3], (ii) driving a stem cell differentiation pathway [4,5], (iii) changing a macrophage phenotype [6], and (iv) modulating the cell membrane potential [7], which play a key role in cytoskeleton remodeling and cell division-related proteins, mainly influencing bacterial cell division [8].

In slow-growth conditions, the timing of cell division in *Escherichia coli* is determined by at least two processes: genome replication/segregation and an interdivision process. The latter relates cell division timing to the size at birth [9]. These cell division processes are endogenous, and it seems unlikely that they can be remotely interfered with from the outside to speed up or stop the division of bacteria. Here, we present proof-of-concept experiments and a theoretical model that demonstrate how the division of *E. coli* Nissle 1917 (EcN) can be controlled by a nonuniform static magnetic field and biogenic magnetic nanoparticles (BMNs) resident inside these probiotic bacteria. Specifically, these BMNs are subjected to the action of magnetic gradient forces, allowing to control not only the cell behavior but also the rates of cell division and growth. Below, we show how the EcN bacterium’s shape dynamics and division rate can be modified by the gradient MF of a specific spatial distribution.

In fact, in the mitosis stage, the separation of a cell into two new ones is caused by internal mechanical forces of the order of 10 pN [10] and coincidentally, the magnetic gradient forces are of the same order of magnitude or even larger [11]. However, to reach a proper interference of the magnetic forces with the mechanical forces to control the division process, it is necessary to know the precise spatial magnetic field distribution and the bacterium’s position in it. This key point is, however, absent in most studies on the biological effects of magnetic fields. 

Bacteria live in noisy environments and, therefore, cell division is a stochastic process with respect to the shape dynamics and timing of division [12,13]. Nevertheless, an application of a static MF can squeeze variance and skewness of division times and the cell sizes in their statistical distributions. This is possible due to the correlation between the mechanical and magnetic forces inside a bacterium. Several remote approaches to influence cell division have been proposed in previous studies. A 27 T static magnetic field was found to orient a spindle in both CNE-2Z and RPE1 human cells [14]. Localized magnetic gradient forces applied to cell-internalized magnetic nanoparticles have been shown to modify DNA orientation and division of HeLa cells [15]. A possibility of preventing tumor cells from dividing by magnetic pressure was theoretically demonstrated in [11].

We have already mentioned that bacteria use mechanical forces to divide [16], and the magnetic gradient forces can either assist or disrupt bacteria division. To justify this assumption, we should address two questions. What is the order of the magnitude of the internal forces of bacteria? How large do the magnetic forces need to be and how should they be directed? 

Magnetotactic bacteria (MTB) are very sensitive to external magnetic fields because they are known to be carriers of native magnetic nanoparticles—known as BMNs [17,18]. Biogenic magnetic nanoparticles have been the object of intensive research since 1975, when they were first detected in MTB. MTB exhibit magnetotaxis, or movement in response to magnetic fields, which makes them migrate along geomagnetic field lines. In later studies, BMNs were found in many organisms of all three domains, i.e., prokaryotes, archaea, and eukaryotes. Formed in the process of biomineralization, crystalline BMNs are nanocrystalline forms of antiferromagnets or ferrites, such as magnetite, maghemite, or greigite [19]. From the point of view of magnetic properties, there are two types of BMNs, with and without remanent magnetization; those without remanent magnetization include antiferromagnetic BMNs and ferrite BMNs in the superparamagnetic (SPM) phase, while remanent magnetization is displayed by ferrite BMNs in single-domain (SD) and multidomain (MD) states. The SPM state is a form of magnetic ordering which appears in small ferromagnetic or ferrimagnetic nanoparticles; magnetization of SPM nanoparticles can randomly change direction under the influence of temperature. The SD state of magnetic nanoparticles refers to the state of a ferromagnet or ferrite in which the magnetization does not vary across the magnet. The MD state of magnetic nanoparticles is related to sufficiently large ferromagnetic or ferrite nanoparticles having several magnetic domains (a magnetic domain is a region within a magnetic material in which the magnetization is in a uniform direction). 

As a rule, ferrite BMNs, in addition to being very sensitive to the applied MF, are permanent nanomagnets, showing remanent magnetization in a wide temperature range and generating a stray magnetic field, which in their vicinity is approximately four orders of magnitude stronger than the magnetic field of the Earth. The ferrite, i.e., magnetite and maghemite, biogenic magnetic nanoparticles are typically detected in multicellular organisms, while biogenic magnetic nanoparticles of bacterial origin are characterized by wide diversity of strain-specific physical and chemical properties [19]. Such BMNs are known to play an important role in magnetoreception [20,21,22,23,24], but they could play a more direct role in the division of bacterial cells exposed to magnetic fields. Due to the magnetic dipole–dipole interaction, the magnetic nanoparticles self-assemble into 1D chains at the cell membrane [25]. BMN chains are best placed on cellular membranes [26]. For the first time, biogenic nanoparticle chains were revealed in MTB [17] that intracellularly make nano-dimensioned greigite or magnetite single-domain (SD) magnetosomes [17,27]. Magnetosomes are membranous structures present in MTB [28,29,30,31]. They contain iron-rich magnetic nanoparticles that are enclosed within a lipid bilayer membrane [28]. Each magnetosome can often contain tens of magnetite or greigite crystals that form a chain. Recent research has shown that magnetosomes are invaginations of the inner membrane and not freestanding vesicles [31]. The magnetosome chain consists of some additional structures, e.g., a cytoskeletal filament and a magnetosomal matrix [29]. These components are highly ordered with various magnetosome-associated proteins. The covering and surrounding of BMN in non-MTB and in archaea are less studied and differ in many ways from BMN in MTB. As a rule, BMN in non-MTB and in archaea are not enclosed in magnetosome vesicles [30]. In archaea, in which the surroundings of BMN have been adequately investigated, magnet-sensitive inclusions have been found, which are surrounded by a homogeneous matrix with organic (mainly protein) components [30]. The absence of vesicles or a similar organic matrix consisting of protein filaments around magnetite BMN [31] has also been observed in human cells. 

Importantly, cell division in magnetotactic bacteria splits the magnetosome chains in half [32]. Below, we show that, in EcN bacteria, the magnetic forces can assist the intracellular mechanical forces to split the BMN chains. Biomineralization of BMN is genetically programmed in MTB [33]. Most of the proteins involved in BMN biomineralization in MTB are encoded in the magnetosome island (MI) [33]. These proteins include the MamA, MamB, MamM, MamE, and MamO proteins, without which the process of BMN biomineralization in MTB is impossible. This means that the MamA, MamB, MamM, MamE, and MamO proteins form an essential set of proteins for the biomineralization of BMN in MTB. Other MI proteins of MTB are regulatory proteins that regulate the shape, size, and quantity of BMN in the cell, the formation of magnetosome vesicles, and the chain-like organization of BMN [34]. There are homolog proteins to all essential proteins for BMN biomineralization in MTB in the human proteome [34] and in the proteomes of animals, plants, fungi, and other multicellular and some unicellular nonmagnetotactic organisms with magnetosensitive inclusions [35,36]. There is an idea of the existence of a single genetically programmed mechanism of BMN biosynthesis in multicellular and many unicellular organisms [35,37]. This mechanism relies on protein homologs of all proteins essential for BMN biomineralization in MTB [35,37].

Furthermore, a bioinformatics analysis using comparative genomics methods in the BLAST program of the US National Center for Biotechnology (NCBI) is performed to determine whether EcN has natural magnetically driven properties, i.e., whether EcN is a producer of BMNs. The EcN proteome was aligned with the amino-acid sequences required for the biomineralization of Mam proteins of the magnetotactic bacterium *Magnetospirillum gryphiswaldense* MSR-1, for which BMN biomineralization has been studied in detail at the genetic level. The bioinformatics research of Mam protein homologs in proteomes of other organisms is useful for searching protein candidates for experimental verification of participation of these homologs in BMN biomineralization. One such successful prediction of the papers [35,37,38] about participation of PEX5 human protein (homologs of mamA) in BMN biomineralization was confirmed experimentally [39]. A review of experimental investigations [19] summarized the information about the indispensable set of Mam proteins and regulatory proteins of magnetotactic bacteria. MamK protein is important for the formation of chains of magnetic nanoparticles in magnetotactic bacteria. However, fundamental differences have been revealed in the process of magnetosome chain formation in different magnetotactic bacteria. “In AMB-1, empty magnetosomes are arranged as a chain prior to magnetite formation. In contrast, magnetosomes of MSR-1 are more dispersed and require magnetic interactions to form a chain” [40]. The review of the experimental data about the observation of biosynthesis of magnetic nanoparticles and their physicochemical properties in non-magnetotactic bacteria [36] shows that the presence of mamK homolog in bacterium proteome is not obligatory for the formation of chains. The chains of biogenic magnetic nanoparticles can be formed due to magnetic interactions similarly to the formation of chains of artificial magnetic nanoparticles at the membrane of a microorganism [25]. Magnetic nanoparticles can self-arrange in the form of agglomerates, chains of nanoparticles, chains of their agglomerates, or more complicated structures. The static configuration of a system of magnetic nanoparticles can correspond to any of many possible local minima of the system free energy. The chains of magnetic nanoparticles represent the most possible patterns at the first stage of their self-arrangement (when the concentration of the nanoparticles is not high) [41]. The self-arrangement of magnetic nanoparticles starts from randomly spatially distributed nanoparticles; then, the self-assembled nanostructures of magnetic nanoparticles evolve into net-like nanostructures passing through the chain and Y-like structures [41]. The complicated patterns of self-arrangement of magnetic micro- and nanoparticles (including chains or stripes of magnetic particles or of their agglomerates, hexagonal lattices of agglomerates of magnetic particles, and others) are also the object of intense research in the field of magnetism and magnetic materials [42], especially in ferrofluids [43]. This is why typically both biogenic and nonbiogenic magnetic nanoparticles and their conglomerates can form chains at the cell membrane due to self-organization process [25,36,40]. There are more examples such as the loop-like structures and double chains of magnetic nanoparticles or of their conglomerates, observed in plant samples when growing plants on soils with the addition of artificial magnetic nanoparticles [44].

The goal of this work is twofold: (i) to provide a condition of cultivation of bacteria to increase their magnetophoretic mobility, focusing on EcN bacteria at high spatiotemporal precision under gradient magnetic field; (ii) to influence the division and growth of bacteria remotely and controllably. We use the magnetic gradient forces generated by our setup to manipulate EcN bacteria and accelerate cell division during mitosis. In a gradient magnetic field, EcN bacteria with BMNs (natural magnetic properties) move without artificial magnetic labeling and reach the area with the highest value of magnetic field induction.

With remote magnetic control of dividing, growing, and delivering EcN to an organ or specific site, these probiotic bacteria can be used for biomedical applications, e.g., for gene delivery and cancer treatment. 

Since *E. coli* are an integral part of the human gastrointestinal flora, they are considered as an alternative for targeted drug/gene delivery through the gut when using gene therapy [45]. There are examples of integration of *E. coli* with magnetic nanoparticles and nanoliposomes loaded with photothermal agents and chemotherapeutic molecules *Escherichia coli* with ~90% efficiency [46]. The magnetic properties of EcN bacterial complexes [46] [47] and the ability to focus them with MFs make them perspective magnetic bacterial vectors in strategies to achieve targeted gene delivery. It is important for cancer treatment that, unlike *S.* Typhimurium and other bacteria that colonize tumors with necrosis, *E. coli* Nissle 1917 has a higher ability to target a tumor, as it mainly propagates in the area between the necrotic and hypoxic regions of tumors [48,49,50]. This guarantees the penetration into the hypoxic areas of the drug, which is loaded into EcN. An additional advantage of EcN is that the cell membrane of EcN can directly interact with the adaptive immune system and, therefore, reduce inflammation [51,52]. Moreover, serum sensitive lipopolysaccharide (LPS) of the EcN membrane provides rapid removal of this strain from normal organs and tissues. Therefore, EcN-like cells with a specific immune regulatory system and a capability of targeting hypoxic sites have potential applications for transporting chemotherapeutic agents to the depth of the tumor site [53].

## 2. Materials and Methods

The *E. coli* Nissle 1917 strain was used in the study. Bacteria *E. coli* Nissle 1917 were cultivated under optimal temperature 37 °C. To cultivate bacteria, a microtube of Eppendorf type was used. The following cultivation media and conditions of cultivations were used for investigations:

(1) Standard cultivation medium—meat–peptone agar (ST). The cultivation medium meat–peptone agar contained (mass/volume) 10 g/L peptone (a source of organic nitrogen), 3 g/L yeast extract (to ensure the content of water-soluble vitamins, carbohydrates, nitrogen, and salts), 10 g/L microbiological agar (to give the mixture hardness), 5 g/L sodium chloride (to give a mixture of sodium proportions similar to those found in the cytoplasm of most organisms), and distilled water (to serve as a transport medium for various substances of agar, with pH adjusted to neutral (is 6.8) at 25 °C.

Manufacturer’s company: “Farmaktiv” LLC (Kyiv, Ukraine), Ukraine manufacturer’s website: http://farmaktiv.com.ua/index.php/ua/ (accessed on 30 October 2022).

Preparation: First, 28 g of nutrient agar powder (MPA) was dissolved in 1 L of hot distilled water, and stirred for complete dissolution of the powder particles. Next, it was cooled to 50 °C and poured into appropriate containers. Then, it was sterilized by autoclaving at 1.1 atm (121 °C) for 15 min. The thermostat was used to maintain the temperature range $\pm 0.5{\;}^{{}^{\circ}}C$ of the working volume during cultivation of bacteria.

(2) Standard cultivation medium—meat–peptone agar with addition of iron chelate (32 mg of iron chelate solution per 1 L of meat–peptone agar) (ST + Fe).

The chemical formula of iron chelate is C_10_H_12_FeN_2_O_8_·Na, CAS number: 15708-41-5, molar mass: 367.05 g/mol.

**Figure 1:**
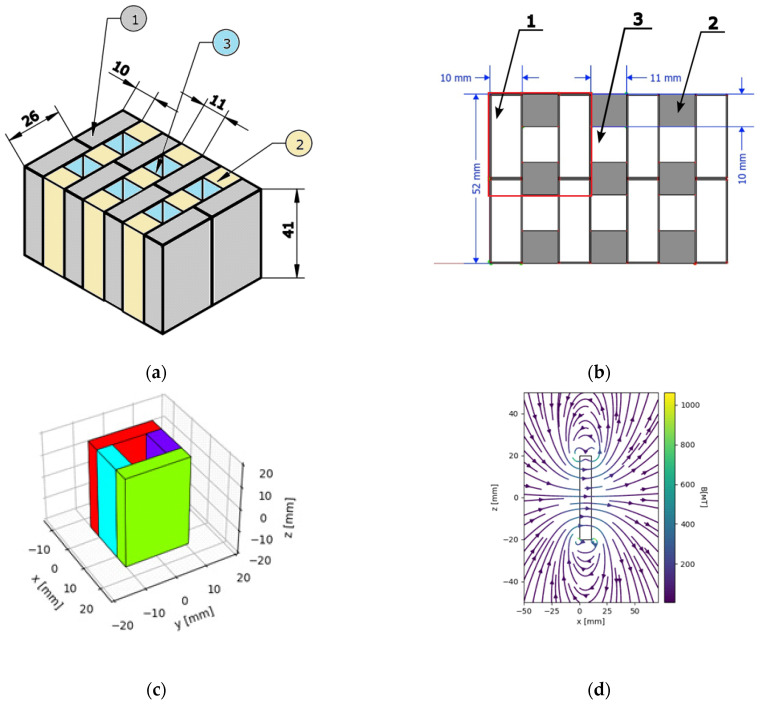
Magnetic system for cultivation of EcN bacteria. In (**a**,**b**), 1—permanent magnet, 2—magnetic circuit, and 3—working volume of the magnetic system for cultivation of the EcN. The magnetic field flux density was measured to be a maximum of 0.15 T inside the working volume of the magnetic system. The measurement was carried out by means of a Magnetic Field Flux Density Meter Ш1-8 with Hall-type sensor. The size of the magnetic field sensor (Magnetic Field Flux Density Meter Ш1-8) is 6 mm. Therefore, the specified sensor can measure the magnetic field flux density averaged on the scale of the characteristic dimensions of the sensor at the center of the working volume (3). The red frame shows the unit cell of the magnetic system used for calculation of both the magnetic field flux density distribution and the magnetic field gradient distribution in the working volume of the magnetic system. (**c**,**d**) Unit of the magnetic system and the spatial distribution of the MF within it. The magnetization of the magnets is directed along the x-axis. The magnetic field flux density at the central area is 150 mT.

**Figure 2:**
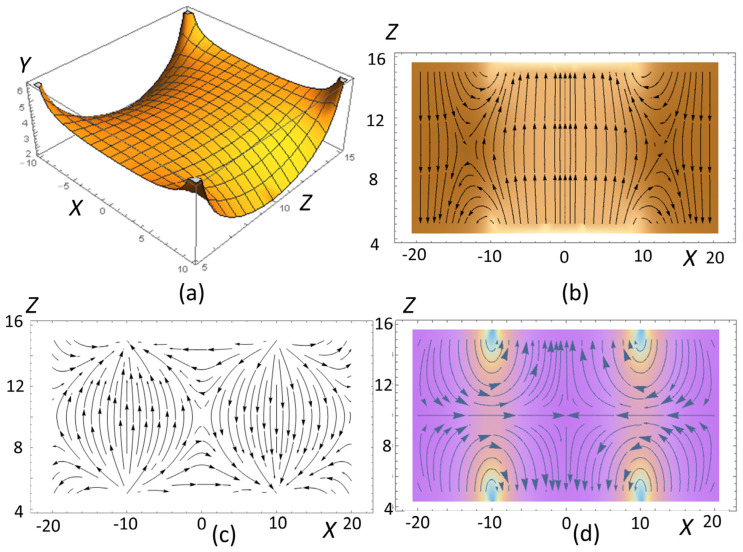
Spatial magnetic field and gradient distributions within a magnetic unit of the bacteria cultivation system: (**a**) 3D plot of the magnetic field strength modulus, *H/M* vs. *x*- and *z*-coordinates (both in mm); (**b**) vector field of ***H*** vs. *x*- and *z*-coordinates in the plane y = 0; (**c**) vector field of *dH/dx* vs. the *x*- and *z*-coordinates in the plane y = 0; (**d**) vector field of *dH/dz* vs. the *x*- and *z*-coordinates in the plane y = 0.

(3) Standard cultivation medium—meat–peptone agar under influence of an external magnetic field. The microtube was placed inside the working volume of a system of permanent magnets (Figure 1 and Figure 2) for cultivation under the influence of an external magnetic field (ST + Fe).

(4) Standard cultivation medium—meat–peptone agar with addition of iron chelate solution (32 mg of iron chelate solution per 1 L of meat–peptone agar) and under influence of an external magnetic field. The microtube was placed inside the working volume of a system of permanent magnets (Figure 1 and Figure 2) for cultivation under the influence of an external magnetic field (ST + MF + Fe).

The inoculating loop was used for collecting the bacteria from a solid culture medium and for making the cell suspensions to study the magnetophoresis of clusters of *E. coli* Nissle 1917 cells. The inoculating loop was made from nickel–chromium alloy, which is resistant to constant heating and cooling. The inner diameter of the loop was 3 mm. A culture grown in Eppendorf containers was collected using the loop and dissolved in water.

**Figure 3:**
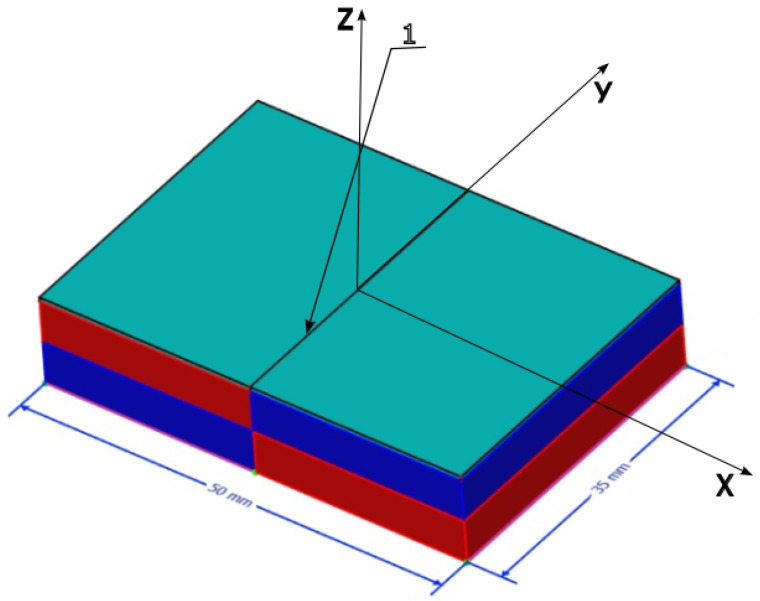
Two permanent magnets generate a gradient magnetic field. The magnetic gradient reaches its maximum just above the contact surface (1).

**Figure 4:**
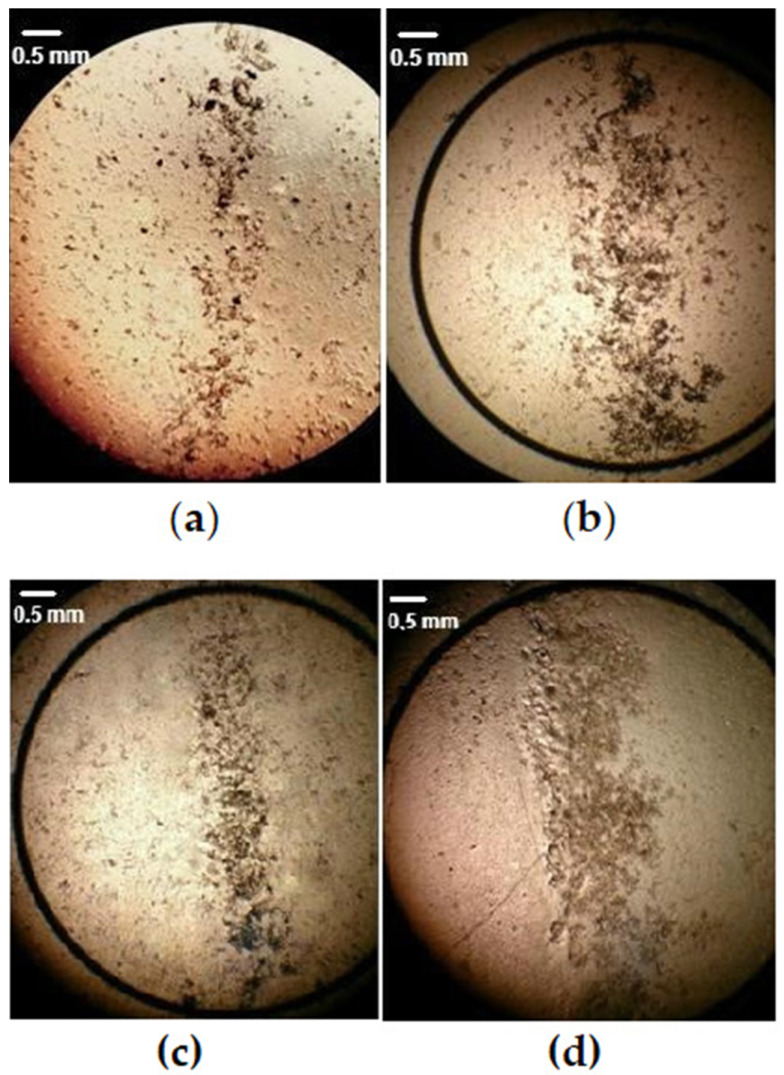
Focusing of EcN bacteria in a gradient MF above the contact surface of the two permanent magnets shown in Figure 3: (**a**) bacterial cells cultivated on standard medium (control); (**b**) bacterial cells cultivated on standard medium with the addition of chelates; (**c**) bacterial cells cultivated on standard medium under the influence of external MF with magnetic field flux density 1500 Oe (0.15 T); (**d**) bacterial cells cultivated on a standard medium with the addition of chelates under the influence of external MF with magnetic field flux density 1500 Oe (0.15 T).

To study the magnetophoresis of bacteria, 0.02 mL of each suspension with a concentration of 3 × 10^7^ cells/mL was applied to a cover glass (0.2 mm thickness), which was placed above the contact surface of the system of two permanent magnets (Figure 3). The band of bacterial clusters (Figure 4) was formed in the inhomogeneous MF. The bandwidth was studied by means of optic microscopy with photo and video capture. The size of the bandwidth and bacterial cluster size distributions were measured using the Gwyddion free software [54].

Additional study of bacteria *E. coli* Nissle 1917 was carried out using the scanning probe microscopy NT-MDT in atomic force mode (AFM) and magnetic force mode (MFM). The physical principles of the dynamic MFM mode are given in detail at the website of the producer of the scanning probe microscopy NT-MDT [55]. The change in resonant properties of the vibrating system cantilever and sample is registered in the dynamic MFM mode caused by magnetic dipole–dipole interaction of the ferromagnetic tip of the cantilever and the sample. In this case, the colormaps of MFM images represent the phase of the cantilever oscillation [55]. The phase of the cantilever oscillation characterizes the force of the magnetic dipole–dipole interaction of the ferromagnetic tip of the cantilever and the sample [55]. During the measurement, the “two-pass” technique was used which consists of the microscopy of two types: AFM and MFM. We used silicon cantilevers MFM_LM with tip curvature radius < 40.0 nm (material—single-crystal silicon, N-type, 0.01–0.025 Ω·cm, antimony-doped; chip size—3.4 × 1.6 × 0.3 mm; reflective side coating—Al; tip coating—CoCr). Low-Moment magnetic high-resolution silicon AFM cantilever MFM_LM series are specially designed to work in magnetic AFM mode (MFM) with low coercive magnetic samples. MFM_LM probes have a thinner magnetic coating than ordinary magnetic tips. Thanks to their own low magnetic fields and low moment, MFM_LM probes allow decreasing the probe influence on the sample while scanning. Due to special defensive layers which prevent the ferromagnetic tip coating from oxidizing, all MFM probes have a long shelf life, and the small tip curvature radius enables obtaining good high-resolution images. The typical resonant frequency of the probes is 70 kHz (guaranteed range 47–90 kHz), and the typical force constant is 3 N/m (guaranteed range 1–5 N/m). The cantilever has an Al reflective side coating to increase the laser signal.

## 3. Results

### 3.1. Magnetic Properties and Magnetophoresis of E. coli Nissle 1917

The standard parameters were taken into account in the alignments to evaluate the homology of the amino-acid sequences of the EcN proteins with the MSR-1 proteins [56]. *E*-number is an indicator of the statistical significance of the alignment; Ident (*I*) is the percentage of identical amino acid residues in the pairwise alignment of a given protein sequence; Length is the alignment length and functions of the proteins under study. The alignment results show that EcN is a BMN producer (Table 1). 

In this research, we considered only proteins of magnetotactic bacteria without which the biomineralization of magnetic nanoparticles is impossible (indispensable set of mam-proteins represented in Table 1). We did not consider the regulatory proteins that are conserved proteins found virtually in all magnetotactic bacteria because biomineralization of magnetic nanoparticles can take place in magnetotactic bacteria without these proteins.

**Figure 5:**
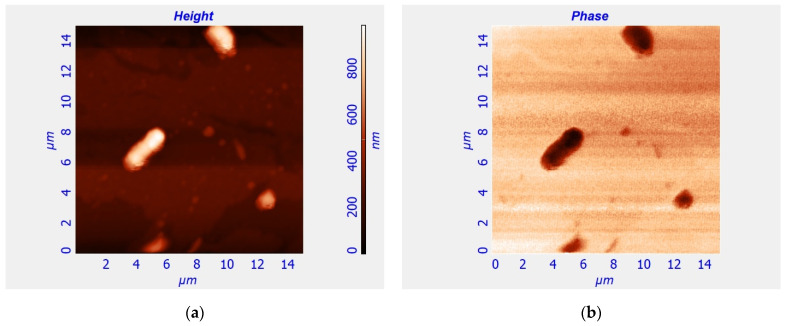
AFM (**a**) and MFM (**b**) images of EcN cells cultivated on standard medium (control). The **left** image represents AFM of EcN cells, i.e., topography of the surface of EcN cells at the cover glass. The color bar for **left** image shows the height in nanometers illustrating the shape and size of EcN cells. The **right** image represents MFM (of the same EcN cells as in **left** image) in the dynamic MFM mode. The abrupt change of color between inside and outside regions of EcN cells in the **right** image means that the force of magnetic interaction of the magnetic tip of the cantilever with the EcN cells differs in a step-like manner from the force of interaction with the diamagnetic cover glass.

The presence of BMN in EcN cells was illustrated using atomic force microscopy (AFM) and magnetic force microscopy (MFM) methods (Figure 5).

The MFM Image of Figure 5 shows that an uneven distribution of BMNs in the form of chain-like clusters (the term “BMN chain” will, unless specified otherwise, refer to the “chain-like clusters”) of different sizes is observed inside the EcN cells, similar to the results of [30], where a study of BMN in Escherichia coli VKM B-126 was performed using transmission electron microscopy. This result, according to the results of bioinformatics analysis (Table 1) and the research [36] and experimental studies of our work (Figure 5), as well as the data reported in [30], indicates that, in the studied EcN cells, BMNs constitute quasi-parallel chains. 

**Figure 6:**
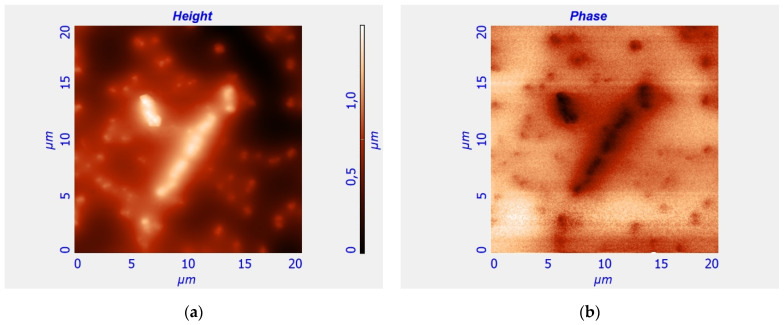
AFM (**a**) and MFM (**b**) images of EcN cells cultivated on medium supplemented with iron chelate. The **left** image represents the AFM of EcN cells, i.e., the topography of the surface of EcN cells at the cover glass. The color bar for the **left** image shows the height in micrometers illustrating the shape and size of EcN cells. The **right** image represents the MFM (of the same EcN cells as in **left** image) in the dynamic MFM mode. The abrupt change in the colors between the inside and outside regions of EcN cells in the right image means that the force of magnetic interaction of the magnetic tip of the cantilever with the EcN cells differs in a step-like manner from that the force of interaction with the diamagnetic cover glass.

An aqueous solution of iron chelate (32 mg/mL) was added to intensify the process of BMN formation and to enhance the magnetically driven properties of EcN cells in standard EcN growing media such as meat–peptone agar and meat–peptone broth. The EcN culture was cultivated on a medium supplemented with iron chelate for 2 days under optimal temperature conditions of 37 °C. After that, the morphological characteristics of BMN bacteria were re-evaluated by means of the methods of AFM and MFM (Figure 6). As can be seen from Figure 6, both EcN cells and BMNs inside the EcN cells formed chains after growing on a standard medium with the addition of iron chelate. 

EcN bacteria were cultivated under four different conditions for a detailed study of the possible methods of changing magnetically controlled properties: (a) standard medium, (b) standard medium with the addition of an aqueous solution of iron chelate (32 mg/mL), (c) standard medium under the influence of a gradient external magnetic field, and (d) standard medium with the addition of an aqueous solution of iron chelate (32 mg/mL) under the influence of a gradient MF with the mean induction value of 0.15 T. The magnetic system used for the cultivation of the EcN cells is shown in Figure 1. Samples of suspensions of the four cultures under study were analyzed at the contact surface of the system of two NdFeB permanent magnets (Figure 3).

Figure 5 and Figure 6 show that the bacterial cells of *E. coli* Nissle 1917 contain magnetic nanoparticles but not paramagnetic ions or molecules. To prove that the bacterial cells of *E. coli* Nissle 1917 contain magnetic nanoparticles but not paramagnetic ions or molecules, we performed a series of magnetophoretic experiments (see Movies 1–4 in Supplementary Materials). The measured velocities of magnetophoresis of the *E. coli* Nissle 1917 clusters under the gradient magnetic field generated in the magnetic system (Figure 3) are listed in Table S1 (Supplementary Materials).

Importantly, high values of the magnetophoretic velocities cannot arise from of diluted paramagnetic species in EcN. We estimated (Section 5 of Supplementary Materials) a hypothetical number of paramagnetic ions, atoms, or proteins with paramagnetic centers (such as ferritin-like proteins) that are necessary to provide the velocity of magnetophoresis observed for the clusters of *E. coli* Nissle 1917 (Table S1). The estimates unambiguously show that the magnetophoretic mobility of the *E. coli* Nissle 1917 clusters observed in the present study can be explained only by the formation of magnetic nanoparticles. Summarizing, the results of the magnetophoretic experiments and theoretical estimations allow us to conclude that the magnetic properties of *E. coli* Nissle 1917 are determined by clusters of BMNs inside cells.

### 3.2. EcN Cultivation in the Presence of a Gradient MF

To cultivate bacteria, a microtube of Eppendorf type was used. The microtube was placed inside the working volume of a system of permanent magnets (Figure 1 and Figure 2) for cultivation under the influence of an external magnetic field. 

To study the magnetophoresis of bacteria, 0.02 mL of each suspension with a concentration of 3 × 10^7^ cells/mL was applied to a cover glass (0.2 mm thickness), which was placed above the contact surface of the system of two permanent magnets (Figure 3). The bandwidth (Figure 4) formed in the inhomogeneous MF above the contact surface of the system of two permanent magnets varied significantly for each of the four studied cultures, which characterizes the magnetic interaction of BMN of the bacteria with a gradient magnetic field generated by the magnetic system. The EcN cells cultivated under the influence of an external MF manifested self-arrangement in the chain structures (Figure 6). Such a chain-like arrangement is typical for objects possessing remnant magnetization and interacting as magnetic dipoles. 

**Figure 7:**
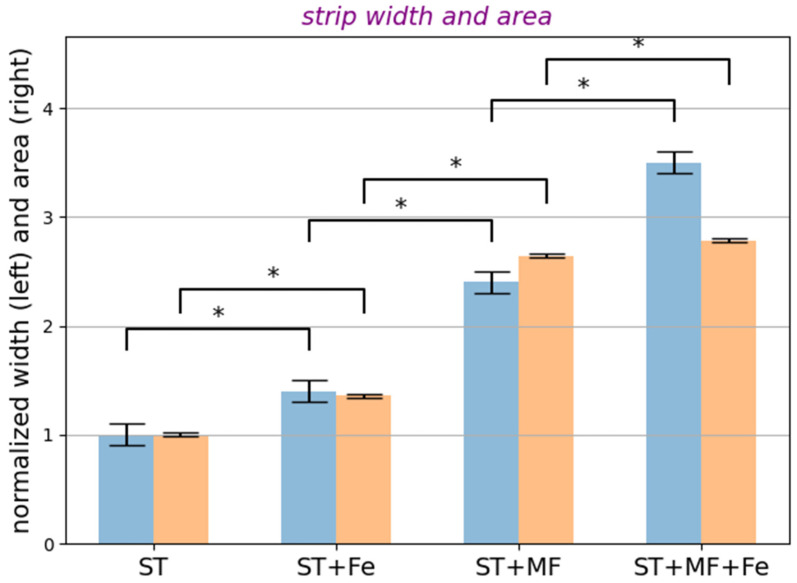
Width and surface area (both normalized to the controls) of the strips, formed by the EcN cell just above the contact surface of the system of two permanent magnets: ST—standard medium (control), ST + MF—standard medium in the external 0.15 T MF, ST + Fe—standard medium with iron chelates, ST + MF + Fe—standard medium with iron chelates in the external 0.15 T MF. The examples of optic images of strips of magnetic focusing of EcN bacteria in a gradient MF above the contact surface of the two permanent magnets are presented in Figure 4. Blue bars represent the mean strip width, while the orange bars are the mean strip area measured using the optic images and the Gwyddion free software [54]. The asterisk (*) means *p* values less than 0.05.

The analysis of bacterial distributions due to their magnetophoretic movement are presented in Figure 7. For this analysis, we used the Gwyddion program [54], which determined the width and surface area of the strips formed by the EcN cell clusters, as well as the average diameters of the EcN cell clusters. Parameters such as the width and length of the strips formed by *E. coli* clusters on the surface of the magnetic system (Figure 3) were used because these parameters increase with the increase in bacterial cluster magnetic susceptibility (see Supplementary Materials).

**Figure 8:**
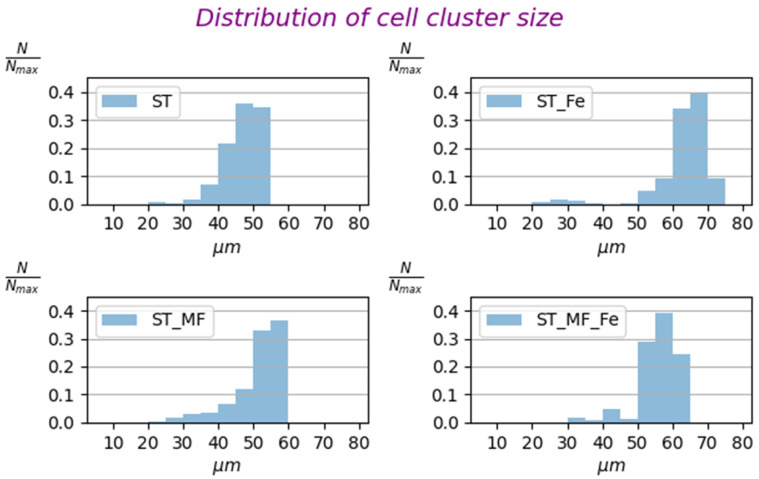
Distributions of the cell cluster sizes of EcN bacteria: *N* is the number of bacterial cell clusters, and *N*_max_ is the total number of bacterial cell clusters analyzed. ST—standard medium (control), ST + Fe—standard medium with iron chelates, ST + MF—standard medium in the gradient 0.15 T MF, ST + MF + Fe—standard medium with iron chelates in the gradient MF of 0.15 T. The *x*-axis refers to the size of bacterial cell clusters in the suspension used to study magnetophoretic velocity.

As seen from Figure 8, the MF application slightly changed the cluster size distributions of EcN. However, the statistical analysis (see Supplementary Materials) reveals that the cluster size was slightly greater for ST + MF—standard medium in the external 0.15 T MF and ST + MF + Fe—standard medium with iron chelates in the external 0.15 T MF in comparison with ST—standard medium (control). This is explained by two competing mechanisms affecting the mean cluster size of bacteria in the MF presence. First, the applied magnetic field tends to line up EcN bacteria in the direction of vector of the magnetic induction, which leads to an increase in the lateral magnetic repulsive forces between the bacteria, thereby expanding the cluster width. Second, the dipole–dipole attraction of BMN chains of neighboring bacteria lined up one behind the other makes the cluster denser.

### 3.3. Model and Predictions of Accelerating Cell Division in Gradient Magnetic Fields of a Special Spatial Configuration

To account for magnetic gradient forces acting on *E. coli* Nissle 1917 bacteria, below, we calculate and map the magnetic field distributions in both magnetic systems used: for culturing bacteria (Figure 1) and for bacteria focusing (Figure 3). 

**Figure 9:**
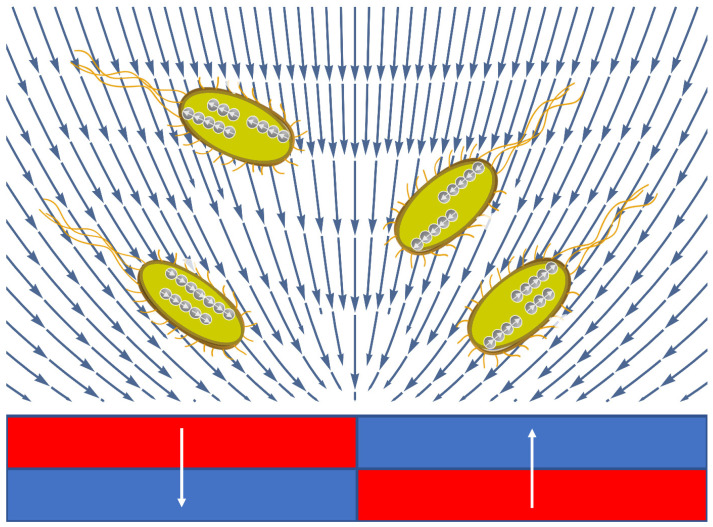
Calculated spatial distribution of ∇B above the magnets. The blue arrows show the gradient directions, which are the same as the magnetic gradient force directions, while the white arrows represent the magnetization of BMNs in bacteria.

Let us consider a bacterium which is located just above the center of the magnetic system (*x* = 0) as shown in Figure 9. Now, we show that, on the left and on the right, the *x*-components of the forces acting on the BMN chains have the opposite directions.

For these forces, we derived analytical expressions to prove that magnetic gradient forces exerted on BMNs are comparable to intracellular forces and, therefore, are capable of altering the functionality of an individual *E. coli* and cell colony. 

First, we calculated the field distribution near the surface of the magnetic system consisting of two permanent magnets with opposite directions of magnetization (Figure 2a–d). Such a magnetic system generated quite a nonuniform field distribution characterized by a sufficiently large value of the magnetic gradient in the vicinity of the plane separating the magnets (Figure 2c,d).

#### 3.3.1. Mapping Magnetic Field Distribution

**Figure 10:**
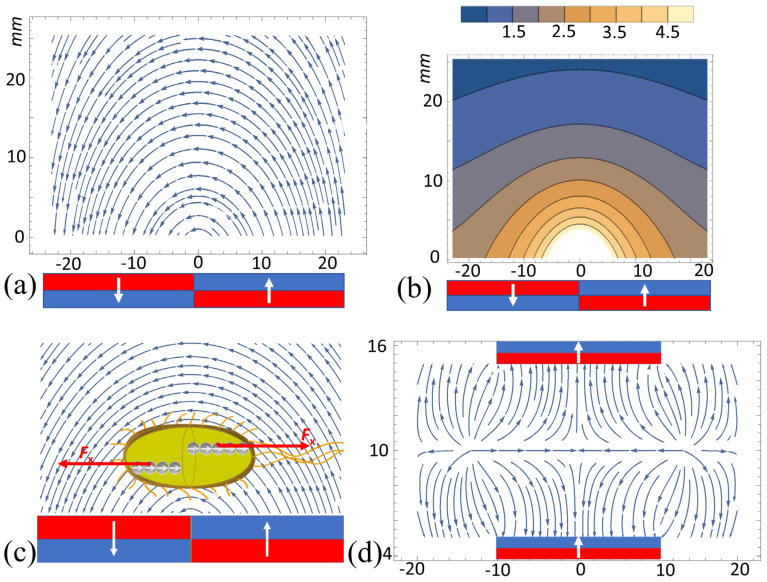
Magnetic field distributions and bacterial division. (**a**) Vector field of the magnetic field strength, ***H*** (the blue arrows). (**b**) Spatial distribution of its modulus above the two magnets of the lateral half size; *a_1_* = 23 mm and thickness 2*a_3_* = 10 mm. The field was calculated in the plane 0.2 mm above the top of the magnets. The white arrows show the directions of the magnetizations of the magnets. The legend shows the values of |***H***|/*M*. (**c**) Bacterial division by the magnetic gradient forces and the magnetic field distribution (blue arrows); the red arrows are the *x*-components of the forces acting on the BMN chains in the magnetic system shown in Figure 10. (**d**) Vector field of the magnetic gradient in unit of the cell incubator shown in Figure 1 (the horizontal and vertical axes are the x- and z-coordinates, respectively).

The system consisted of two strips magnetized as shown in Figure 9. The geometrical parameters of the magnets are provided in the caption of Figure 10. 

The magnetic force acting on a nanoparticle with a magnetic dipole moment is proportional to the field gradient, i.e., *F∝*∇*B* (where *B* is magnetic induction). We calculated the spatial distributions of the magnetic field and its gradient generated by this magnetic system.

**Figure 11:**
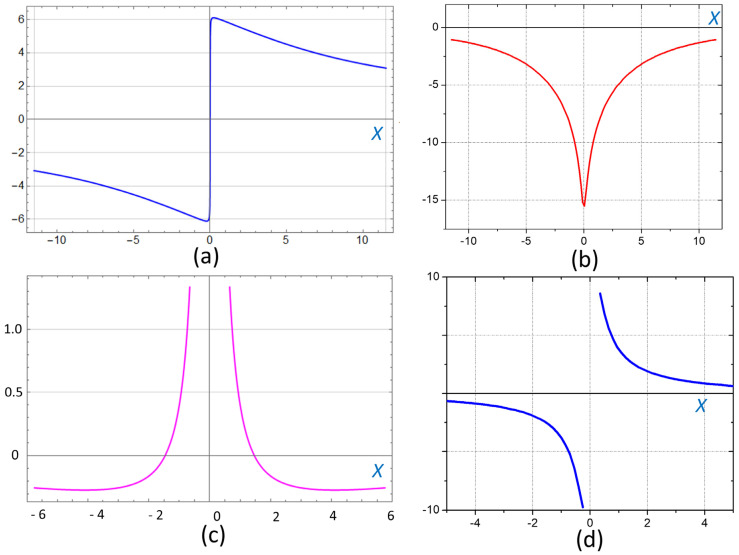
Calculated magnetic field strength components and their derivatives: (**a**) *H_z_/M*, (**b**) *H_x_/M*, (**c**) (*dH_z_/dx*)*M*^−1^, and (**d**) (*dH_x_/dx*)*M*^−1^ (both derivatives are in mm^−1^), versus the *x-* coordinate (in mm) The calculations were made in the plane 0.2 mm above the surface of the magnets. Note, *x* = 0, the origin of the coordinate system, is in the center of the magnetic system.

For a long uniformly magnetized stripe, the magnetic field strength components are expressed as follows [57,58]:

(1) $$\frac{H_{z}}{M_{2}}=-2\left(Ar\mathrm{ctg}\left(\frac{a_{1}-x}{a_{3}-z}\right)+Ar\mathrm{ctg}\left(\frac{a_{1}+x}{a_{3}-z}\right)+Ar\mathrm{ctg}\left(\frac{a_{1}-x}{a_{3}+z}\right)\;+Ar\mathrm{ctg}\left(\frac{a_{1}+x}{a_{3}+z}\right)\right),$$

(2) $$\frac{H_{x}}{M_{2}}=ln\left(\frac{\left({\left(a_{1}+x\right)}^{2}+{\left(a_{3}-z\right)}^{2}\right)\left({\left(a_{1}-x\right)}^{2}+{\left(a_{3}+z\right)}^{2}\right)}{\left({\left(a_{1}-x\right)}^{2}+{\left(a_{3}-z\right)}^{2}\right)\left({\left(a_{1}+x\right)}^{2}+{\left(a_{3}+z\right)}^{2}\right)}\right),$$

where 2*a_1_* and 2*a_3_* are the magnet dimensions along the X- and Z-axes, and *H_z_* and *H_x_* are the magnetic field strength components. Below, we assume that ***M*** = (0, 0, *M*), where *M* is the remanent magnetization of the magnets. The calculated vector field of ***H*** and its modulus |***H***| distributions are shown in Figure 10 and Figure 11. In the central area, just above the top of the magnets between their centers, *H_x_* < 0 (Figure 11b). 

The magnetic field strength components *H_x_/M* and *H_z_/M*, as well as their derivatives (gradients) (*dH_x_/dx*)*M*^−1^ and (*dH_z_/dx*)*M*^−1^ as functions of the x-coordinate, calculated near the top surface of the two magnets (Figure 10), are shown in Figure 11. Below, we show that these magnetic gradients determine the magnetic forces acting on BMN chains in bacteria. 

#### 3.3.2. Magnetic Forces Exerted on BMN Chains

We calculated the magnetic force acting on a BMN chain in the gradient magnetic field defined by Equations (1) and (2) and shown in Figure 9 and Figure 10. The magnetic gradient force acting on a magnetic dipole is given by $\vec{F}=\left(\vec{p}\;\vec{\nabla }\right)\vec{B}$, where ***p*** is the magnetic dipole moment of a BMN chain, ***B*** is the magnetic induction, ∇ is the differential operator nabla, ***B*** = μ_0_***H*,** and μ_0_ = 4π 10^−7^ NA^−2^ is the vacuum permeability. Assuming the chain’s magnetic moments to be parallel to the x-direction, i.e., ***p*** = (*p_x_*,0,0), the projections of the force ***F*** on the x- and z-coordinates read as

(3a) $$F_{x}=\mu_{0}p_{x}\frac{dH_{x}}{dx},$$

(3b) $$F_{z}=\mu_{0}p_{x}\frac{dH_{z}}{dx}.$$

As Equation (3) and Figure 11d show, the magnetic gradient force *F_x_* > 0 for d*H_x_*/d*x* > 0 and *F_x_* < 0 for d*H_x_*/d*x* < 0. For the given orientation of the dipole moment, the z-component of the magnetic force (Equation (3b)) also changes its direction according to the derivative d*H_z_*/d*x* (Figure 11c). 

For a body of volume *V* and susceptibility χ placed in a magnetic field of strength ***H***, the magnetization (the magnetic moment per unit volume) obeys ***M_s_*** = ∑***p****/V* = χ***H***, which is valid up to very high magnetic fields. By combining Equation (3) with the previous formulas and using *p* = *M_s_*V, one can arrive at

(4a) $$F_{x}=\mu_{0}M_{s}V\frac{dH_{x}}{dx},$$

(4b) $$F_{z}=\mu_{0}M_{s}V\frac{dH_{z}}{dx},$$

where *V* = *N*4π*R*^3^/3 is the BMN chain volume. 

The magnetic gradient forces act on bacteria in two ways: (i) on BMN chains located inside a bacterium, as given by Equation (4); (ii) on the bacterium body due to the difference in the susceptibilities of bacterium and medium; ∆χ = *χ_b_* − *χ_m_*, where $\chi_{b}$ is the bacterium magnetic susceptibility, and $\chi_{m}$ is the magnetic susceptibility of the medium. 

For a diamagnetic body, the magnetic gradient force is defined by

(5) $$\vec{f}=V_{b}\Delta \chi \frac{B\nabla B}{\mu_{0}},$$

where *V_b_* is the bacterial volume. Bacteria and typical cell media are generally considered to be diamagnetic; both *χ_b_* and *χ_m_* are negative. However, due to the presence of BMN chains, a bacterium is less diamagnetic than the surrounding medium, i.e., *Iχ_b_I* < *Iχ_m_I*, which means that the BMN chain volume is too small to overcome the dominant diamagnetism of a bacterium. In this case, Δχ > 0; therefore, the force ***f*** is parallel to the magnetic gradient (Equation (5)). Thus, the bacteria are attracted to the areas with the highest magnetic field, as illustrated in Figure 9. Note that Figure 11 shows the spatial distribution (the vector field) of the gradient of the modulus of ***B*** as calculated from Equations (1) and (2). It can be seen that the forces attract bacteria to the bottom of the central area of the magnetic system. This qualitatively corresponds to the observations depicted in Figure 4 (see the Supplementary Materials). Thus, due to the magnetic gradient forces (Equation (5)), bacteria are concentrated in the middle plane of the magnetic system.

#### 3.3.3. Mechanism of *E. coli* Division in an MF Requires a Special Spatial Configuration of the Field

The dependence of *dH_x_*/*dx* as calculated from Equations (1) and (2) is shown in Figure 11d. Thus, since *p_x_* > 0 and *dH_x_*/*dx* > 0 for *x* > 0 and *dH_x_*/*dx* < 0 for *x* < 0 (Figure 11d), the horizontal force components (Equation (3)) have the opposite signs on the left and right parts of the system: F_x_ > 0 for *x* > 0 and *F_x_* < 0 for *x* < 0 (Figure 10 and Figure 11). 

The horizontal magnetic force exerted on a chain consisting of *N* of biogenic magnetic nanoparticles of the radius *R*. In this case *V* = *N*4π*R*^3^/3, and Equation (4) reads as

(6) $$F_{x}=\frac{4\pi }{3}NR^{3}\mu_{0}M_{s}\frac{dH_{x}}{dx},$$

where *M_S_* is the saturation magnetization of Fe_3_O_4_, which is about 80 emu/g (*M_s_*  =  412  kA/m). To estimate a force value with Equation (6), we need to know the gradient value, *dH_x_*/*dx*. This quantity is plotted in Figure 11d and infinitely grows on approaching *x* = 0. Indeed, in the vicinity of the two magnet boundaries (*x*/*a_1_* << 1), *H_x_* can be approximated as follows [59]:

(7) $$H_{x}\left(x\right)=4Mln\left(\frac{a_{1}}{x}\right).$$

From Equation (7), we can arrive at the field gradient:

(8) $$\frac{dH_{x}}{dx}=-\frac{4M}{x}.$$

The dependence given by Equation (8) is exactly the same as that shown in Figure 11d. Thus, the planar gradient value infinitely grows on approaching the boundary *x* = 0. Then, the magnetic force exerted on the dipole chain is

(9) $$F_{x}=\frac{16\pi }{3x}NR^{3}\mu_{0}M_{s}M=\frac{16\pi }{3x}NR^{3}B_{r}M_{s},$$

where *B_r_* = μ_0_*M* is the magnetic induction at the top magnet surface, e.g., for a NdFeB magnet μ_0_*M* ≈ 1–1.2 T. As seen from Equation (8), on approaching the boundary between the magnets, the magnetic force can be large enough; *F_x_* tends to infinity when *x* → 0. The force component *F_z_(x)* manifests similar behavior on approaching the boundary between the magnets because d*H_z_*/d*x* infinitely grows when *x* → 0 (Figure 3). Obviously, we cannot operate with an infinite force. Instead, using Equation (9), we estimate the magnetic force magnitude for the distance *x* = 1 mm from the boundary between the magnet (*x* = 0), *N* = 20, and *R* = 20 nm. Assuming that a bacterium is just on the magnet top (*B_r_* = 1 T), and inserting *M_s_* = 412 kA/m into Equation (9), one can obtain *F_x_* ≈ 20 pN. Of note, this force reaches 200 pN at the distance *x* = 0.1 mm. 

We calculated the magnetic gradient force (Equation (9)) exerted on BMN chains of bacteria in the magnetic system shown in Figure 3. The magnetic system where the EcN bacteria were cultivated (Figure 1) was assembled from permanent magnets with the same magnetic characteristics and had similar length scales. Thus, during cultivation, EcN bacteria were exposed to a gradient MF with the gradient of the same order of magnitude as calculated for the magnetic system shown in Figure 3. This allowed us to conclude that, during cultivation, the EcN bacteria were subjected to the magnetic gradient forces of the order (10–100) pN as estimated above. 

Note, in magnetotactic bacteria, to break a magnetosome chain, one should apply a force of 10–50 pN [60,61,62] and forces of the same order of magnitude normally produced during cell division. For example, using magnetic nanoparticles with 50 pN magnetic gradient forces, HeLa cell division was achieved in [15]. Moreover, the magnetic forces exerted on the BMN chains of bacteria are as large as the typical bacteria traction forces which are hundreds of pN [63].

Thus, cell division can be altered by magnetic forces (Equation (9)) because this force is exerted on BMN chains bound to the intracellular components and, therefore, may interfere (assist or counteract) with the endogenous forces causing bacterial division.

#### 3.3.4. Magnetic Gradient Force Changes the Timescale of Bacterial Division 

It is envisioned that, in MTBs, magnetosome chain localization at the midcell might be controlled by early-assembling components of the divisomal complex, perhaps via direct interaction with the magnetosome filament [62]. Thus, it is natural to assume that, in EcN bacteria, BMN chains are connected to the divisomal complex. In this case, the magnetic gradient force can directly change the DNA topology and facilitate chromosome segregation at the time of division. It is known that the rate of division of *E. coli* is higher when more nutrients are available. However, nutrients cannot change the length of periods C and D. On the other hand, during the D period, the mechanical forces play an important role in cell division. In a gradient MF of the special configuration shown in Figure 1 and Figure 2, the magnetic gradient forces assist the endogenous mechanical forces to break the bacterial BMN chains in two parts, thereby shorteingn the D period. As shown in the previous section, the magnetic forces can be one order of the magnitude larger that the mechanical force causing cell division.

The growth rate of bacteria is

(10) $$r={\left(T_{B}+T_{C}+T_{D}\right)}^{-1},$$

where *T*_B_, *T*_C_, and *T*_D_ are the durations of the B, C, and D periods, respectively. Of note, the B, C, and D period durations are not fixed but vary depending on the growth rate. In particular, the B period is extremely variable in length. Under fast growth conditions, the B stage is skipped entirely [64]. 

In *E. coli* K-12 MG1655 strain, under slow growth conditions, the B, C, and D periods take 8, 54, and 34 min, respectively [64,65]. For this case, from Equation (11), an estimate gives the growth rate *r* = 0.01 min^−1^. Unfortunately, in the literature to date, it seems that, for the magnetosensing *E. coli* Nissle 1917, the *T*_B_, *T*_C_, and *T*_D_ periods are still unmeasured. Such measurements are highly demanded because cell division in magnetosensing bacteria has two specific features: (i) to divide, a bacterium must split BMN chains in half [32]; (ii) a bacterium bends its internal BMN chains to weaken it before cell division [61,62]. 

Notably, the time needed for a relocation of magnetosomes in dividing magnetotactic bacteria extends the D period of the cell cycle. A gradient magnetic field of a specific configuration (shown in Figure 9 and Figure 10) facilitates both processes: (i) bacterium bending to minimize its energy in the MF, and (ii) BMN chains breaking by the magnetic gradient forces (Figure 10c). Moreover, bending the BMN chain is the way to weaken the magnetic dipole–dipole forces holding the magnetic chain. The mechanism of BMN chain positioning and division in *Magnetospirillum gryphiswaldense* was studied by electron microscopy in [62], where it was shown that a specific adaptation is required to overcome the magnetic dipole–dipole interactions between separating daughter chains. Obviously, this adaptation takes additional time and prolongs the cell cycle and D period; for example, in *Magnetospirillum gryphiswaldense*, the entire cell cycle was completed after 260 min with the main phases: 120–180 min of extensive elongation, followed by a second phase lasting another 40 min, in which cells gradually constricted at midcell, with the acute bending and snapping proceeding very quickly in less than 7 min [62]. Thus, the conclusion that can be drawn from this experiment is that, in magnetosensing bacteria, the entire cell cycle and the D period are longer than those in nonmagnetic bacteria.

As mentioned before, magnetic gradient forces favor the magnetosome chain division via two mechanisms: bacterium bending and chain breaking. Specifically, this MF action shortens the D period, thereby increasing the growth rate of magnetosensing bacteria. The *T*_D_ period depends on the magnetic gradient in the area where a bacterium is dividing. 

**Figure 12:**
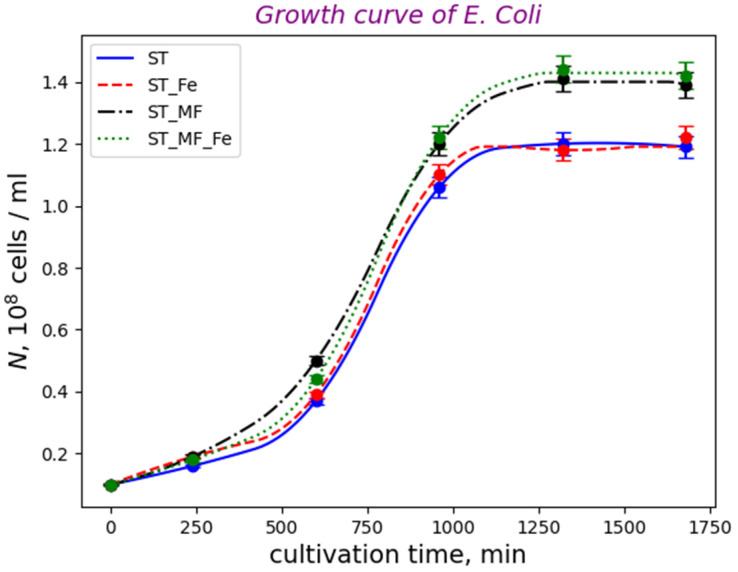
Growth curves of *E. coli* Nissle 1917 cultivated under different conditions: ST—standard medium (control), ST + Fe—standard medium with iron chelates, ST + MF—standard medium in the gradient 0.15 T MF, and ST + MF + Fe—standard medium with iron chelates in the gradient 0.15 T MF (*p <* 0.05). Here, *N* is the number of cell clusters per ml of cell suspension.

Unfortunately, in the literature, measurements of the *T*_B_, *T*_C_, and *T*_D_ periods in *E. coli* Nissle 1917 are absent; therefore, we cannot calculate the growth rate from Equation (10). However, on the basis of the measured time dependences of the bacterial number (Figure 12), we can calculate the maximal growth rates for EcN cultures cultivated in different conditions: in medium with the addition of iron chelate in the presence of the MF; in a standard environment in the presence of the MF; in medium with the addition of iron chelates; in a standard environment (control).

#### 3.3.5. Experimental: Static Gradient MF Accelerates Growth of *E. coli* Nissle 1917

To monitor the effect of the chelating medium and the magnetic field on the growth, cultivation of *E. coli* Nissle 1917 was carried out for 28 h with periodic measuring of the number of living cells by counting in a counting chamber device originally designed and usually used for counting blood cells.

In Figure 12, we selected the time interval where bacteria grow exponentially (steady state) and solved the problem of linear regression for the *ln*(*N*) dependences vs. time for each curve in this time interval using the scipy and numpy packages of python programming language. These dependences are linear in the time range from 0 up to 1000 min. On the linear parts of the *ln*(*N*) curves, the growth rate is maximal [66] and the time dependence of the bacteria number obeys

(11) $$N\left(t\right)=N_{0}e^{\frac{t\;ln2}{\tau }}=N_{0}e^{rt},$$

where *N(t)* is the concentration of bacteria vs. time, *N_0_ = N(0)*, and *t* and *r* are the doubling time and growth rate, respectively. The growth rate and mean doubling time in a steady state are related through the equation *r* = *ln*2/*τ*.

For each curve shown in Figure 12, the maximal growth rate was determined by a linear fitting with *ln*(*N) = a + rt*, where *a* and *r* are the fitting constants. The python programming language and its package *scipy* were used for the calculation of *a*, *r*, and standard error of the estimated slope *r*, under the assumption of residual normality and the Pearson correlation coefficient. The obtained growth rates and doubling time were as follows: *r_ST_* = (0.00246 ± 0.00014) min^−1^ (correlation coefficient = 0.997) and *τ* = 282 min for EcN bacteria cultivated on the standard environment (control), *r_ST+MF_* = (0.00260 ± 0.00004) min^−1^ (correlation coefficient = 0.9997) and *τ* = 267 min for EcN cultivated on the standard environment in the presence of the gradient MF, *r_ST+Fe_* = (0.00244 ± 0.00013) min^−1^ (correlation coefficient = 0.997) and *τ* = 284 min, for EcN cultivated on the medium with the addition of iron chelates, and *r_ST+Fe+MF_* = (0.00260 ± 0.00007) min^−1^ (correlation coefficient = 0.999) and *τ* = 267 min for EcN cultivated on the medium with the addition of iron chelate in the presence of the gradient MF. In comparison, for “nonmagnetic” Escherichia coli, the growth rate was 0.016 min^−1^ and the cell doubling time was *τ* = 43.5 min for strain NCM 3722 delta-motA [67], and the maximal growth rate was 0.0219 min^−1^ for *E. coli* K-12 BW25113 [68]. For magnetosensitive bacteria *Magnetospirillum gryphiswaldense* containing magnetosome chains, the mean doubling time was *τ* ≈ 260 min [62]. Thus, in comparison with nonmagnetic *E*. coli bacteria, the growth rate was ~5 times higher. This is in line with the abovementioned fact that, in magnetosensitive bacteria, the D period is prolonged. However, the addition of iron chelate did not have any significant effect on cultivation processes (Figure 12) and the growth rate of EcN. 

Thus, in both cases (for EcN bacteria cultivated on the standard environment and EcN cultivated on the medium with the addition of iron chelates), the gradient magnetic field increased the bacteria growth rate by 5.7%. Importantly, as expected, adding iron did not change the growth rate.

The effect of increasing the growth rate was small, which is explained below. For magnetosensitive bacteria, the upper limit of the increase in the growth rate can be estimated as follows: in the cell cytoplasm, characteristic velocities (*v*) of macromolecules and their complexes are proportional to intracellular mechanical forces (*F*). If a gradient MF is applied, magnetic gradient forces assist intracellular forces to drive the cell separation. Since intracellular and magnetic gradient forces (*F_m_*) are on the same orders of magnitude, the force driving cell division is doubled (*F_0_ = F + F_m_* ≈ 2*F*); therefore, the characteristic velocity is also doubled (in Stokes flow *v*~*F_0_*). Then, assuming that the mitosis time is inversely proportional to the characteristic velocity (∆t~1/*v*), we obtain that magnetic forces shorten the mitosis time by half. Lastly, keeping in mind that, in bacteria, the relative duration of the mitotic phase is 10–15% of the cell-cycle time [69,70], one can conclude that magnetic forces can decrease the mitosis duration twofold, i.e., squeezing them down to 5–7%. This implies that the time needed to complete segregation in the anaphase and telophase was shortened by the magnetic gradient forces. According to Equation (11), a 5–7% decrease in the cell-cycle period would lead to a growth rate increase of 5–7%. In our experiment, the MF application increased the EcN growth rate by (*r*_ST+MF_ − *r*_ST_)/*r*_ST_ ≈ 6%. Thus, there is good agreement between the theoretical estimation and experimental result. 

The obtained results reveal that division acceleration offers the possibility of interfering with the help of a magnetic field in the process of division of magnetically sensitive bacteria. Indeed, the presence of BMNs in bacteria complicates the process of their division and, consequently, reduces the growth rate of the individual bacterium and colony. The gradient magnetic field makes it easier to break BMN chains and divide bacteria, thus slightly increasing the growth rate.

## 4. Discussion

Here, we performed proof-of-concept experiments and proposed a theoretical model to reveal and explain a new effect: magnetically accelerated cell division and growth. We showed the possibility of interfering with the processes of cell division and growth using gradient MFs of specific spatial configuration.

The analysis using comparative genomics methods, along with atomic and magnetic force microscopy, revealed that *Escherichia coli* Nissle 1917 is a BMN producer. *Escherichia coli* Nissle 1917 is a known vector for drug delivery and gene therapy, and its natural magnetic properties allow its magnetically controlled administration to lesion sites and/or organs in a human body. In this view, our findings are important for using *E. coli* Nissle 1917 cell culture for magnetically targeted drug and gene delivery. 

Our approach to changing the cell machinery by regulating the division time and growth rate with MFs is also interesting for chemical production of probiotic bacteria. The magnetogenetic-based cell division regulation strategy can greatly improve the efficiency of microbial cell factories and medical applications of magnetosensitive bacteria.

## Figures and Tables

**Table 1:** Alignment of the proteins of the Mam group of *M. gryphiswaldense* MSR-1 and the proteome of the bacterium EcN.

The Strain of the Microorganism	*E*-Number (*І*, %)				
	Proteins of *Magnetospirillum gryphiswaldense* MSR-1				
	MamA	MamB	MamM	MamO	MamE
*Escherichia coli* Nissle 1917	0.001 23.86%	$5\times 10^{-37}$ 30.54%	$2\times 10^{-29}$ 29.89%	$3\times 10^{-9}$ 29.70%	$1\times 10^{-35}$ 40.38%

## Data Availability

No new data were created.

## Supplementary Materials

The following supporting information can be downloaded at https://www.mdpi.com/article/10.3390/cells12020315/s1: Statistical analysis of the distributions of the cluster sizes of EcN bacteria. Movie 1. Magnetophoresis of EcN bacteria grown in standard medium; Movie 2. Magnetophoresis of EcN bacteria grown in standard medium with iron chelates; Movie 3. Magnetophoresis of EcN bacteria grown in standard medium in the gradient 0.15 T MF; Movie 4. Magnetophoresis of EcN bacteria grown in standard medium with iron chelates, in the gradient 0.15 T MF; Table S1: Average velocity if magnetophoresis of the clusters of *E. coli* Nissle 1917 under the gradient magnetic field of the system of permanent magnets [71,72,73,74,75,76,77,78].
