# Supplementary material for: Gradient Magnetic Field Accelerates Division of E. coli Nissle 1917
Source: Cells. 2023 Jan 14;12(2):315. doi: 10.3390/cells12020315 (PMC9857180; doi:10.3390/cells12020315)
Supplement: Supplementary file 1 [file cells-12-00315-s001.zip › cells-2042594-proof supplementary.pdf]

Supplementary Materials

Gradient Magnetic Field Accelerates Division of *E. coli* Nissle 1917

Svitlana Gorobets [1], Oksana Gorobets [1,2], Iryna Sharai [1,2], Tatyana Polyakova [3] and Vitalii Zablotskii [3,4,*]

[1] Faculty of Biotechnology and Biotechnics (S.G.), Faculty of Physics and Mathematics (O.G. and I.S.), National Technical University of Ukraine "Igor Sikorsky Kyiv Polytechnic Institute", 03056 Kyiv, Ukraine
[2] Institute of Magnetism of the National Academy of Sciences of Ukraine and Ministry of Education and Science of Ukraine, 03142 Kyiv, Ukraine
[3] Institute of Physics of the Czech Academy of Sciences, Na Slovance 1999/2, 182 00 Prague, Czech Republic
[4] International Magnetobiology Frontier Research Center (iMFRC), Science Island, Hefei 230000, China
* Correspondence: zablot@fzu.cz; Tel.: +420-266-05-2436

1. Magnetic Systems for the Cultivation and Focusing of EcN Bacteria

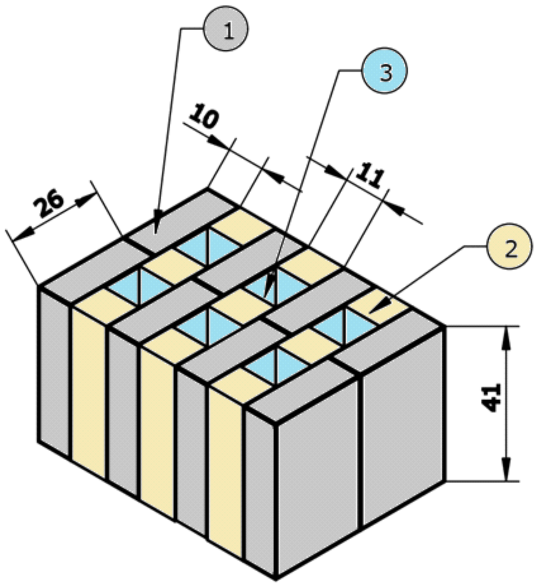

a)

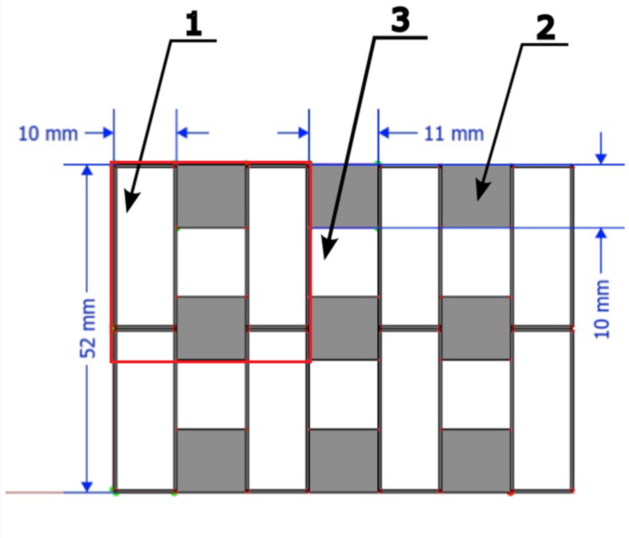

b)

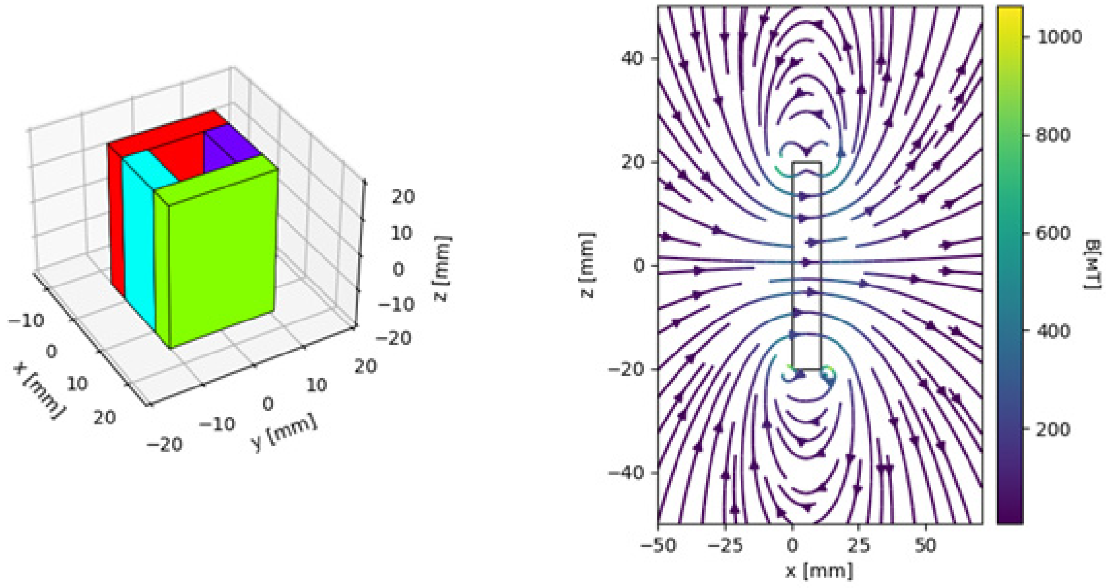

c) d)

Figure S1. Magnetic system for cultivation of EcN bacteria. In figures *a* and *b*, 1 – permanent magnet, 2 – magnetic circuit, 3 – working volume of the magnetic system for cultivation of the EcN. The magnetic field flux density was measured to be maximum 0.15 T inside the working volume of the magnetic system. The measurement was carried out by means of Magnetic Field Flux Density Meter Ш1-8 with Hall-type sensor. The size of the magnetic field sensor (Magnetic Field Flux Density Meter Ш1-8) is 6 mm. Therefore, the specified sensor can measure the magnetic field flux density averaged on the scale of the characteristic dimensions of the sensor at the center of working volume 3. The red frame shows the unit cell of the magnetic system used for calculation of both magnetic field flux density distribution and distribution of gradient of magnetic field in a working volume of the magnetic system. (*c* and *d*): unit of the magnetic system and the spatial distribution of the MF within it. The magnetization of the magnets is directed along the *x*-axis. The magnetic field flux density at the central area is 150 mT.

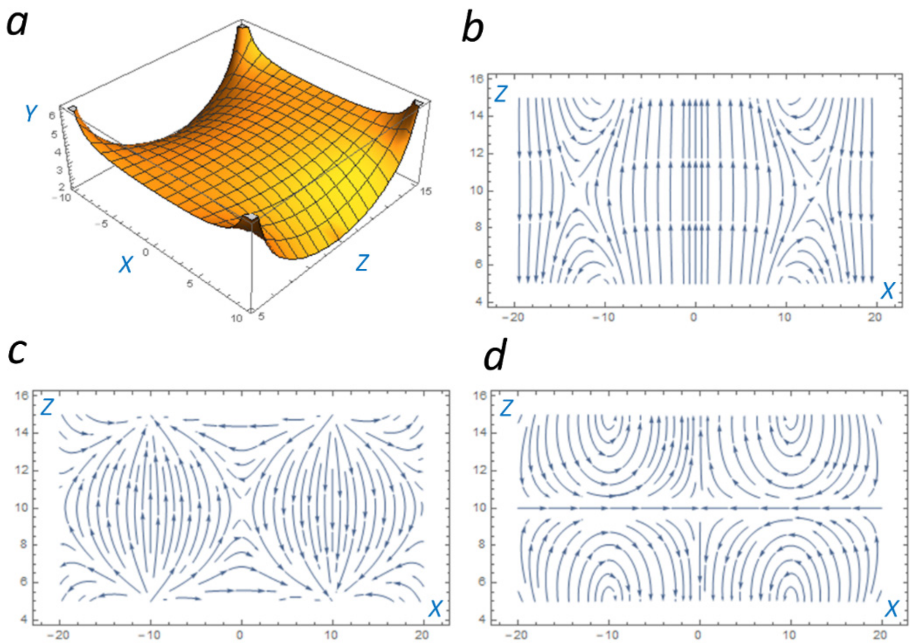

Figure S2. Spatial magnetic field and gradient distributions within a magnetic unit of the bacteria cultivation system: *a*) 3D plot of the magnetic field strength modulus, H/M vs x and z coordinates (both in *mm*), *b*) vector field of $\boldsymbol{H}$ vs the x- and z- coordinates in the plane y=0, *c*) vector field of dH/dx vs the x- and z- coordinates in the plane y=0, and *d*) vector field of dH/dz vs the x- and z- coordinates in the plane y=0.

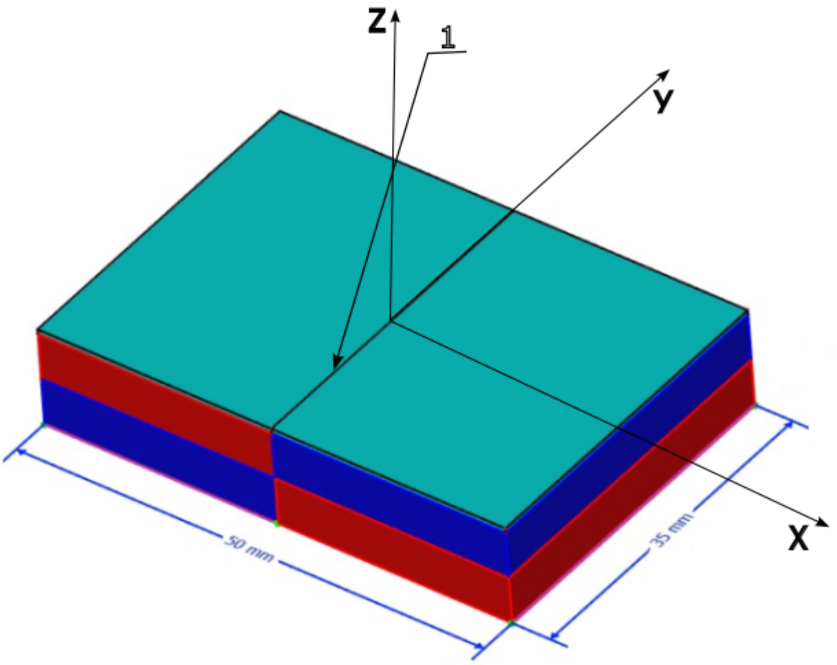

Figure S3. Two permanent magnets generate a gradient magnetic field. The magnetic gradient reaches its maximum just above the contact surface (1).

2. Focusing EcN Bacteria with a Gradient Magnetic Field Generated by Two Magnets

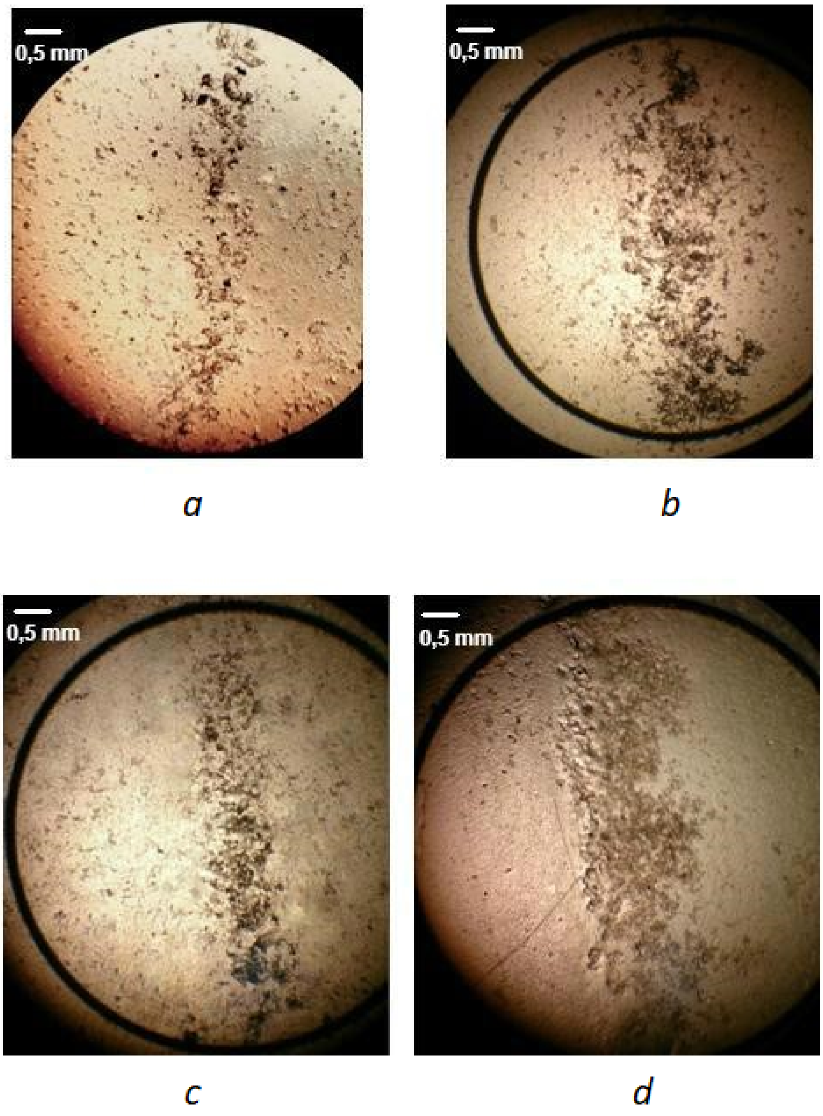

Figure S4. Focusing of EcN bacteria in a gradient MF above the contact surface of the two permanent magnets shown in Fig. S3: *a*) bacteria cells cultivated on standard medium (control); *b*) bacteria cells cultivated on standard medium with the addition of chelates; *c*) bacteria cells cultivated on standard medium under the influence of external MF with magnetic field flux density 1500 Oe (0.15 T); *d*) bacteria cells cultivated on a standard medium with the addition of chelates under the influence of external MF with magnetic field flux density 1500 Oe (0.15 T).

3. Statistical Analysis of the Distributions of the Cluster Sizes of EcN Bacteria

The Gwyddion program was used to measure diameters of EcN cell cluster size distributions (Fig. 4) on the basis of optic microscopy images of EcN cell clusters. The mean and maximum diameters of EcN cell clusters (Fig. 4) cultivated under the influence of an external constant MF are 1.24-1.26 times larger than for the control, and the maximum diameter of the EcN cell clusters differs slightly from the control for samples, cultivated with the addition of iron chelates into the medium (Fig. 4). The Welch's t-Test statistic turns out to be less than zero and the corresponding p-value is less than 0.05. Here the p-value is less than 0.05 hence we could reject the null hypothesis of the test and the conclusion that the difference between the mean cluster diameters of control group and all other three groups (ST+Fe, ST+MF, ST+MF+Fe) of data (Fig. 4) is quite significant. Besides the negative t-value means that the mean of cluster diameter in control (ST- standard medium) was significantly smaller than the mean of all other three groups of data (ST+Fe,

ST+MF, ST+MF+Fe). The Wasserstein distance was calculated also between control distribution in Fig. 4 (ST) and all three distributions in Fig. 4 (ST+Fe, ST+MF, ST+MF+Fe). The three calculated Wasserstein distances represent the average distance between each point of control distribution (ST) and the corresponding point of each of three distributions in Fig. 4 (ST+Fe, ST+MF, ST+MF+Fe). The calculation revealed that the Wasserstein distances are the same as the absolute value of differences between the means of these distributions. The calculation of statistical significance of the results was carried out using build-in methods of scipy package of Python programming language.

4. Velocities of Magnetophoresis of the Clusters of *E. coli* Nissle 1917 under the Gradient Magnetic Field of the System of Permanent Magnets

Table S1. Average velocity if magnetophoresis of the clusters of *E. coli* Nissle 1917 under the gradient magnetic field of the system of permanent magnets (Fig. S3). .

Conditions of cultivation of *E. coli* Nissle 1917	Average velocity, mm/s
ST	0,7
ST+Fe	1,6
ST+MF	1
ST+MF+Fe	1,9

5. Estimation of Number of Paramagnetic Ions, Atoms or Proteins to Provide the Experimentally Observed Velocity of Magnetophoresis for the Clusters of *E. coli* Nissle 1917

Let us estimate the hypothetical number of paramagnetic ions, atoms or proteins with paramagnetic centers (such as ferritin-like proteins) that are necessary to provide the velocity of magnetophoresis observed for the clusters of *E. coli* Nissle 1917 (Table S1). This estimation shows that it is impossible to achieve such a high velocity of magnetophoresis as about 1 mm/s (Table S1) if we take on account contribution of paramagnetic ions, atoms or proteins with paramagnetic centers into the magnetic susceptibility of *E coli* cells according to the literature data about their concentration. Further, this estimation shows that velocity of magnetophoresis would be 3-4 orders of magnitude less than the observed one (Table S1) if we take on account contribution of paramagnetic ions, atoms or proteins with paramagnetic centers into the magnetic susceptibility of *E coli* cells.

The gradient magnetic force acts on the quasi-spherical cluster of *E. coli* under influence of a gradient magnetostatic field ($rot\,\vec{H} = 0$) (Figure 4):

$$\vec{F}_{\nabla B} = \frac{\chi(\vec{B}\nabla)\vec{B}}{\mu_0} V = \frac{1}{2\mu_0}\chi\nabla\vec{B}^2 V, \tag{1}$$

where $\mu_0 = 4\pi \cdot 10^{-7}$H·m^{-1} is vacuum permeability, $\chi = \chi_{cl} - \chi_l$ is the effective magnetic susceptibility of the cluster of *E. coli*, χ_{cl} is the magnetic susceptibility of the cluster of *E. coli*, χ_l is the magnetic susceptibility of liquid in which the cluster is moving, $V = \frac{4}{3}\pi r_{cl}^3$ is the volume of the cluster of *E. coli*, r_{cl} is the radius of the cluster of *E. coli*. The gradient magnetic force is balanced by the viscous damping force

$$\vec{F}_{St} = 6\pi\eta\vec{v}r_{cl}, \tag{2}$$

where η is the dynamic viscosity of liquid, $\vec{v}$ is the magnetophoretic velocity of cluster of *E. coli.* The relation follows from the equality of these forces:

$$\frac{\chi}{\mu_0}\left|\nabla\vec{B}^2\right| r_{cl}^2 = 9\eta v. \tag{3}$$

The magnetic susceptibility of the cluster of *E. coli* can be calculated according to the general formular for magnetic susceptibility of a mixture [1]:

$$\chi_{Cl} = \frac{V_p\chi_p + V_d\chi_d}{V_p + V_d} \tag{3}$$

where V_p and V_d denote the volume of the mixture occupied by paramagnetic and diamagnetic substance correspondingly, whose magnetic susceptibilities are χ_p and χ_d. Taking on account that $V_p \ll V_d \approx V_{cl}$ and $\chi_d \approx \chi_l$ we simplify the last formula

$$\chi_{cl} = \frac{V_p}{V_{cl}}\chi_p + \chi_l. \tag{4}$$

The magnetic susceptibility of the cluster of *E. coli* can be calculated according to Langevin formular at high temperatures:

$$\chi_{cl} = \frac{\mu_0 n g^2 J(J+1)\mu_B^2}{3k_B T} + \chi_l, \tag{5}$$

where n is the number of paramagnetic ions in the unit volume of the cluster of *E. coli* ($\frac{V_p}{V_{cl}} = V_1 n$, where V_1 is an average volume occupied by one paramagnetic atom or ion), μ_B is Bohr magneton, $\mu_0 = 4\pi \cdot 10^{-7}\,\frac{H}{m}$ is the vacuum permeability, $g\sqrt{J(J+1)}$ is the effective number of Bohr magnetons, k_B is Boltzmann constant, T is temperature. The substitution of the expression for χ_{cl} (5) into the equality (3) results in the estimation of concentration of paramagnetic ions in the cluster of *E. coli*:

$$n = \frac{27 k_B T \eta v}{g^2 J(J+1)\mu_B^2 |\nabla \vec{B}^2| r_{cl}^2}. \tag{5}$$

The estimated number of iron ions per *E. coli* cell is

$$N = \pi a b^2 n, \tag{6}$$

where $V_{cell} = \frac{4}{3}\pi a b^2$, a is the long semiaxis, b is short semiaxis of bacterial cell. The values of the universal physical constants are:

$$\mu_B = 9.273 \cdot 10^{-24}\, JT^{-1}$$
$$k_B = 1.38 \cdot 10^{-23}\, JK^{-1}$$

The following parameters were used for estimations according to the formular (6):

$$g\sqrt{J(J+1)} \approx 5 \;\; [2]$$
$$\eta = 10^{-3}\, Pa \cdot s \text{ – dynamic viscosity of water}$$
$$r_{cl} \approx 50\, \mu m \text{ (Fig. 4)}$$
$$T \approx 300\, K$$

$v \approx 1\, mm \cdot s^{-1}$ according to the measurement of magnetophoretic velocity in the present research

$|\nabla \vec{B}^2| \approx 0.5 \cdot 10^2\, \frac{T}{m}$ – typical value for the system of permanent magnets (Fig. S3)

$$2a = 2.83\, \mu m \;\; [3]$$
$$2b = 0.86\, \mu m \;\; [3]$$

The estimations result in the value of $N \approx 10^9$ that is 3-4 orders of magnitude greater than the iron content of *E. coli* ranges from 10^5 to 10^6 atoms per cell, depending on growth conditions [1] [4].

The influence of the level of free radicals on magnetic susceptibility of bacteria is low according to the formular for magnetic susceptibility of a mixture [1] because of low concentration of the free radicals. For example, in fully aerated *Escherichia coli*, the interplay between endogenous production and scavenging enzymes results in a steady-state intracellular concentration of ~0.2 nM O^{2-} [5].

The influence of the level of other paramagnetic atoms (Co, Ni) on magnetic susceptibility of bacteria is also very low according to the formular for magnetic susceptibility of a mixture [1] because of low concentration of the cobalt and nickel in bacterial cells. For example, the divalent cations of cobalt and nickel are essential nutrients for bacteria, are required as trace elements at nanomolar concentrations and are toxic at micro- or millimolar concentrations [6]. Simple estimation shows that the number of ions or free radicals with nanomolar concentration per *E. coli* cell is about several pieces.

Let us consider also an idea that bacterial ferritin of *E. coli* can contribute significantly into it magnetic susceptibility. Three types of ferritin-like proteins in prokaryotes are studied in [7]: classical ferritins (Ftn), which are similar to eukaryotic ferritins, bacterioferritin (Bfr), and "DNA-binding proteins from starved cells" (Dps proteins). Ftn was found to load the highest number of iron atoms (approx. 75 per holomer), followed by Bfr (approx. 44 iron atoms per holomer). Fri bound the lowest amount of iron atoms (approx. 10 per holomer) [7]. When overexpressing the iron-storage proteins, the iron content of the bacteria *E. coli* Nissle 1917 significantly increased (up to 6-fold in Bfr-expressing EcN in culture) [7]. However, important differences are detected in the magnetization behavior for the mammalian ferritin (horse spleen ferritin - HoSF) and the prokaryotic ferritin-like proteins (including bacterioferritin (Bfr) from *Escherichia coli*) [8]. The superparamagnetic behaviour was found in the cores of mammalian ferritin due to uncompensated moments at the surface of the antiferromagnetic ferrihydrite core [8]. The results of the magnetization versus the field measurements indicate that the mineral core synthesized by the prokaryotic ferritin-like proteins lacks magnetic order and behaves like a paramagnet [8]. The results for the HoSF give a magnetic moment per core of ≈340 μ_B [8]. Regarding the prokaryotic samples, the most striking result obtained from the magnetic fits is the very low magnetic moment per particle core, ranging between 6 and 8 μ_B. This is much lower than the value obtained for HoSF (≈ 340 μ_B) [8]. Basically, 6–8 μ_B is an atomic-like magnetic moment (Fe3+ should contribute with 5 μ_B), which indicates that we have a paramagnet from individual uncoupled Fe3+ ions [8]. The following conclusions can be derived from the experimental data [7, 8]:

1. The contribution of magnetic susceptibility of bacterial ferritin into the overall magnetic susceptibility of cluster of *E. coli* is atomic-like and can be described by the formular (5) with the parameter $g\sqrt{J(J+1)} \approx 6-8$ denoting n as the number of ferritin molecules in the unit volume of the cluster of *E. coli*.
2. Even 6-fold increase of iron content of *E. coli* Nissle 1917 (observed in [7]) due to overexpression of Bfr is not sufficient to explain the magnetophoretic mobility of cluster of *E. coli* Nissle 1917 observed in the present research.
3. The magnetophoretic mobility of cluster of *E. coli* Nissle 1917 observed in the present research can be explained by the formation of magnetic nanoparticles.

6. Movies

Movie 1: Magnetophoresis of EcN bacteria grown in standard medium (control). Bacteria move towards the contact surface of the two permanent magnets shown in Fig. S3.

Movie 2: Magnetophoresis of EcN bacteria grown in standard medium with iron chelates. Bacteria move towards the contact surface of the two permanent magnets shown in Fig. S3.

Movie 3: Magnetophoresis of EcN bacteria grown in standard medium in the gradient 0.15 T MF. Bacteria move towards the contact surface of the two permanent magnets shown in Fig. S3.

Movie 4: Magnetophoresis of EcN bacteria grown in standard medium with iron chelates, in the gradient 0.15 T MF. Bacteria move towards the contact surface of the two permanent magnets shown in Fig. S3.

1. Kuchel, P.W.; Chapman, B.E.; Bubb, W.A.; Hansen, P.E.; Durrant, C.J.; Hertzberg, M.P. Magnetic susceptibility: Solutions, emulsions, and cells. *Concepts Magn. Reson. Educ. J.* **2003**, *18*, 56–71.
2. Biedermann, A.R. ; Pettke, T.; Koch, C.B.; Hirt, A.M. Magnetic anisotropy in clinopyroxene and orthopyroxene single crystals. *Am. Mineral.* **1968**, *53*, 406–415.
3. Liu, P.Y.; Chin, L.K.; Ser, W.; Ayi, T.C.; Yap, P.H.; Bourouina, T.; Leprince-Wang, Y. Real-time Measurement of Single Bacterium's Refractive Index Using Optofluidic Immersion Refractometry. *Procedia Eng.* **2014**, *87*, 356–359.

4. Semsey, S.; Andersson, A.M.C.; Krishna, S.; Jensen, M.H.; Massé, E.; Sneppen, K. Genetic regulation of fluxes: Iron homeostasis of Escherichia coli. *Nucleic. Acids. Res.* **2006,** *34*, 4960–4967.
5. Fasnacht, M.; Polacek, N. Oxidative Stress in Bacteria and the Central Dogma of Molecular Biology. *Front. Mol. Biosci*. **2021**, *8*, 671037.
6. Nies, D.H. Resistance to cadmium, cobalt, zinc, and nickel in microbes. *Plasmid.* **1992**, *27*, 17–28.
7. Hill, P.J.; Stritzker, J.; Scadeng, M.; Geissinger, U.; Haddad, D.; Basse-Lüsebrink, T.C.; Gbureck, U.; Jakob, P.; Szalay, A.A. Magnetic resonance imaging of tumors colonized with bacterial ferritin-expressing Escherichia coli. *PLoS One* **2011**, *6*, e25409.
8. García-Prieto, A.; Alonso, J.; Muñoz, D.; Marcano, L.; de Cerio, A.A.D.; Fernández de Luis, R.; Orue, I.; Mathon, O.; Muela, A.; Fdez-Gubieda, M. L. On the Mineral Core of Ferritin-like Proteins: Structural and Magnetic Characterization. *Nanoscale* **2016**, *8*, 1088.
